# Controllable Fast and Slow Light in Photonic-Molecule Optomechanics with Phonon Pump

**DOI:** 10.3390/mi12091074

**Published:** 2021-09-04

**Authors:** Huajun Chen

**Affiliations:** School of Mechanics and Photoelectric Physics, Anhui University of Science and Technology, Huainan 232001, China; hjchen@aust.edu.cn

**Keywords:** photonic molecule, optomechanical system, optomechanically induced transparency, slow light, phonon pump

## Abstract

We theoretically investigate the optical output fields of a photonic-molecule optomechanical system in an optomechanically induced transparency (OMIT) regime, in which the optomechanical cavity is optically driven by a strong pump laser field and a weak probe laser field and the mechanical mode is driven by weak coherent phonon driving. The numerical simulations indicate that when the driven frequency of the phonon pump equals the frequency difference of the two laser fields, we show an enhancement OMIT where the probe transmission can exceed unity via controlling the driving amplitude and pump phase of the phonon driving. In addition, the phase dispersion of the transmitted probe field can be modified for different parametric regimes, which leads to a tunable delayed probe light transmission. We further study the group delay of the output probe field with numerical simulations, which can reach a tunable conversion from slow to fast light with the manipulation of the pump laser power, the ratio parameter of the two cavities, and the driving amplitude and phase of the weak phonon pump.

## 1. Introduction

Cavity optomechanics (COM) systems [1,2], researching the interaction of the photon modes and phonon modes, have obtained great progress in fundamental researches and pragmatic applications in the past few years, including the ground state cooling [3,4,5,6,7], precision measurements [8,9,10,11,12,13,14], and quantum information processing [15,16,17,18]. The radiation pressure forces, in COM systems, induce optomechanical interactions leading to phonon modes, which in turn influence the optical modes and, then, result in remarkable quantum interference effects. At present, lots of important breakthroughs have been reached in COM systems, including phonon lasers [19,20,21,22], squeezing [23,24], entanglement [17,18], optical nonreciprocity [25,26,27], and exceptional point [21,22,28,29]. Another especially amusing phenomenon closely connected to the present paper is optomechanically induced transparency (OMIT), which has been demonstrated in different optomechanical systems [30,31,32,33,34,35,36]. OMIT arises from the destructive interference effect of the two absorption channels of the probe photons and, thus, presents momentous applications in slow light [33,37,38,39], precision measurements [9,13], sensing [40,41,42,43], and the storage of information [44].

Recently, Lü et al. demonstrated that OMIT can also appear in a spinning resonator where the optomechanical system is rotating [45], and then the clockwise and counterclockwise modes of the resonator experience Sagnac–Fizeau shifts. Whereafter, several significant discoveries such as the phonon laser [46], sensing [47], nonreciprocal photon blockade effect [48,49], and optomechanical entanglement generation were identified [50]. Furthermore, if the COM system is driven by an external coherent phonon pump, the OMIT properties [51] will also be influenced significantly. In the investigation of OMIT, the COM is generally driven by one strong pump laser (with the pump frequency $\omega_{p}$) and a weak probe laser (with the probe frequency $\omega_{s}$). If the mechanical mode in the COM system is further driven by a weak coherent mechanical field (with the driving frequency $\omega_{q}$), considering the coupling between the photon modes and phonon modes, another two different optical components in the output field will appear: the first one is induced by the pump and probe laser fields (with frequencies $\omega_{p}\pm n\Omega$) and the second one is induced by the optical pump and phonon pump fields (with frequencies $\omega_{p}\pm m\omega_{q}$), where $\Omega =\omega_{s}-\omega_{p}$, *m* and *n* are integers [52]. Especially, when the condition $\Omega =\omega_{q}$ is met, the destructive instructive quantum interference (or the instructive quantum interference) of the two different optical output components indicates one means to manipulate the properties of the output optical fields in the COM systems.

In this paper, we theoretically study the output probe field in a photonic-molecule optomechanical system which is driven by a weak coherent phonon driving. The numerical simulations indicate that the probe transmission experiences different processes which manifest the OMIT effect by controlling different parametric regimes, such as the decay rate ratio parameter δ of the two optical cavities, the driving amplitudes *f*, and the pump phase $\phi_{m}$ of the phonon driving. The weak phonon pump with a controllable driving amplitude *f* and pump phase $\phi_{m}$, as well as the ratio parameter δ of the two cavities, together lead to an enhanced OMIT, which accompanies the fantastic phase dispersion resulting in an enhanced group delay of the transmitted probe field. With the numerical simulations, the results show that the steerable conversion from the slow light to fast light effect can be easily reached by controlling several parameters.

## 2. Model and Theory

**Figure 1:**
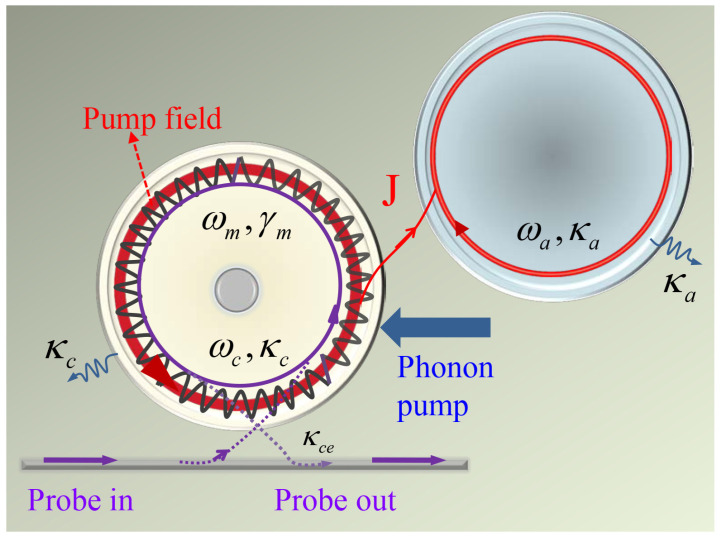
Schematic diagram of the photonic-molecule optomechanics with phonon driving, where an optomechanical cavity c driven by two-tone fields coupled to an auxiliary cavity a with high quality factor.

Figure 1 is the photonic-molecule optomechanical system, including two coupled whispering-gallery-mode (WGM) cavities [21,32,53,54,55], where the optomechanical cavity c with the decay rate $\kappa_{c}$ and frequency $\omega_{c}$ is evanescently coupled to a tapered fibre. The radiation pressure force arriving from the pump laser field coupled into the optomechanical cavity c will induce the radial breathing mode (i.e., the mechanical mode b with frequency $\omega_{m}$ and damping rate $\gamma_{m}$). The coupling between the optical mode c and mechanical mode b is described by an optomechanical coupling rate $g=g_{0}x_{0}$, where $g_{0}=\omega_{c}/R$ as a single-photon coupling rate with *R* is the radius of cavity c, and $x_{0}=\sqrt{\hbar /2M\omega_{m}}$ being the zero-point fluctuation of the mechanical mode with the effective mass *M* of the optomechanical cavity c [32]. The WGM cavity a is an auxiliary cavity with the decay rate $\kappa_{a}$ and frequency $\omega_{a}$ coupled to cavity c with coupling strength *J* [53]. In a frame rotation of the pump field frequency $\omega_{p}$, we can obtain the Hamiltonian of our system as follows [1,21,31,32,56]:

(1) $$\begin{array}{c} \begin{array}{cc} H & =\hbar \Delta_{c}c^{†}c+\hbar \Delta_{a}a^{†}a+\hbar \omega_{m}b^{†}b+\hbar J\left(a^{†}c+ac^{†}\right)-\hbar gc^{†}c\left(b^{†}+b\right)\\ & +i\hbar \sqrt{\kappa_{ce}}\varepsilon_{p}\left(c^{†}-c\right)+i\hbar \sqrt{\kappa_{ce}}\varepsilon_{s}\left(c^{†}e^{-i\Omega t}-ce^{i\Omega t}\right)+2qF_{m}\cos\left(\omega_{q}t+\phi_{m}\right), \end{array} \end{array}$$

where the first three terms are the free Hamiltonian of the cavity modes and mechanical mode, and $\Delta_{c}=\omega_{c}-\omega_{p}$ ($\Delta_{a}=\omega_{a}-\omega_{p}$) is the corresponding cavity–pump field detuning of cavity c (cavity a). We use *c*($c^{†}$), *a*($a^{†}$), and $b(b^{†})$ to describe the annihilation and creation operators of cavity c, cavity a, and mechanical mode b, respectively. The fourth term describes the cavity–cavity interaction with coupling strength *J*, and the fifth term is the optomechanical interaction with coupling strength *g*. The sixth and seventh terms are the input laser fields couple to cavity mode c, and the amplitude of the pump field (probe field) is $\varepsilon_{p}=\sqrt{P/\hbar \omega_{p}}$ ($\varepsilon_{s}=\sqrt{P_{s}/\hbar \omega_{s}}$) with the pump (probe) field power *P* ($P_{s}$), and $\Omega =\omega_{s}-\omega_{p}$ is the pump–probe detuning. For an optical cavity, the decay rate κ includes the intrinsic loss rate $\kappa_{e}$ and extra loss rate $\kappa_{0}$, i.e., $\kappa =\kappa_{e}+\kappa_{0}$, and usually $\kappa_{e}=\kappa_{0}$ [32]. Here, in our system, there were also the relations of $\kappa_{a}=\kappa_{a0}+\kappa_{ae}$ and $\kappa_{c}=\kappa_{c0}+\kappa_{ce}$ for the two optical cavities, where $\kappa_{a0}$ and $\kappa_{c0}$ were still the intrinsic loss rate of the two cavities ($\kappa_{ae}$ and $\kappa_{ce}$ are the extra loss rate of the two cavities), and for simplicity we set $\kappa_{a}=\kappa_{c}$ and $\omega_{c}=\omega_{a}$. The last one gives the mechanical mode b driven by a weak coherent phonon driving, where the parameter $F_{m}$ is $F_{m}=\frac{f}{2\hbar }\sqrt{\frac{\hbar }{M\omega_{m}}}$ with the driving amplitude *f*, the pump phase $\phi_{m}$, and the pump frequency $\omega_{q}=\omega_{s}-\omega_{p}$.

Using the Heisenberg equation of motion and adding the corresponding damping and input noise terms for the cavity and mechanical modes [1,21,32], we then obtained the Langevin equations (LEs) as follows:

(2) $$\partial_{t}c=-\left(i\Delta_{c}+\kappa_{c}\right)c+igcq-iJa+\sqrt{\kappa_{ce}}\left(\varepsilon_{p}+\varepsilon_{s}e^{-i\Omega t}\right)+\sqrt{2\kappa_{c}}c^{in},$$

(3) $$\partial_{t}a=-\left(i\Delta_{a}+\kappa_{a}\right)a-iJc+\sqrt{2\kappa_{a}}a^{in},$$

(4) $$\partial_{t}^{2}q+\gamma_{m}\partial_{t}q+\omega_{m}^{2}q=2g\omega_{m}c^{†}c-2qF_{m}\cos\left(\omega_{q}t+\phi_{m}\right)+\xi ,$$

where $q=b^{†}+b$ means the position operator of the mechanical mode, $c^{in}$ ($a^{in}$) are the input vacuum noises with a zero mean value of the cavity c (a), and ξ is the Langevin force due to the thermal reservoir. Considering the pump field is much stronger than the probe field, we introduced the perturbation theory: $O=O_{0}+\delta O$ (O indicates the operator of c,a, and *q*), i.e., each operator is divided into the steady-state mean value and a small fluctuation with a zero mean value. The steady-state values are determined by $\left(i\Delta^{\prime }+\kappa_{c}\right)c_{0}-iJa_{0}=\sqrt{\kappa_{ce}}\varepsilon_{p}$, $\left(i\Delta_{a}+\kappa_{a}\right)a_{0}+iJc_{0}=0$, and $q_{0}=2g{\left|c_{0}\right|}^{2}/\omega_{m}$, where $\Delta^{\prime }=\Delta_{c}-gq_{0}$.

When we used the mean field approximation $\langle Qc\rangle =\langle Q\rangle \langle c\rangle$ [30], the operators could be replaced by their expectation values, and after being linearized by neglecting nonlinear terms in the fluctuations, the LEs of the expectation values can be obtained as follows:

(5) $$\langle \partial_{t}\delta c\rangle =-\left(i\Delta^{{}^{\prime }}+\kappa_{c}\right)\langle \delta c\rangle +igc_{0}\langle \delta q\rangle -iJ\langle \delta a\rangle +\sqrt{\kappa_{ce}}\varepsilon_{s}e^{-i\Omega t},$$

(6) $$\langle \partial_{t}\delta a\rangle =-\left(i\Delta_{a}+\kappa_{a}\right)\langle \delta a\rangle -iJ\langle \delta c\rangle ,$$

(7) $$\langle \partial_{t}^{2}\delta q\rangle +\gamma_{m}\langle \partial_{t}\delta q\rangle +\omega_{m}^{2}\langle \delta q\rangle =2g\omega_{m}\left(c_{0}^{*}\langle \delta c\rangle +c_{0}\langle \delta c^{†}\rangle \right)-2qF_{m}\cos\left(\omega_{q}t+\phi_{m}\right),$$

which is a set of nonlinear equations containing many frequency components. We defined the ansatz as $\langle \delta O\rangle =O_{+}e^{-i\Omega t}+O_{-}e^{i\Omega t}$ and, by substituting it into Equations (5)–(7), we obtained three group equations with neglecting nonlinear terms in the fluctuations as follows:

(8) $$\begin{array}{c} \left(i\Delta^{{}^{\prime }}+\kappa_{c}-i\delta \right)c_{+}=-igc_{0}q_{+}-iJa_{+}+\sqrt{\kappa_{ce}}\varepsilon_{s},\\ \left(i\Delta_{a}+\kappa_{a}-i\delta \right)a_{+}=-iJc_{+},\\ q_{+}=2g\lambda_{1}\left(c_{0}^{*}c_{+}+c_{0}c_{-}^{*}\right)+F_{m}\lambda_{1}e^{-i\phi_{m}}. \end{array}$$

Solving the equations, we obtained:

(9) $$c_{+}=\frac{igc_{0}\Lambda_{2}^{*}F_{m}\lambda_{1}e^{-i\phi_{m}}+\left(\Lambda_{2}^{*}-2ig^{2}\lambda_{1}{\left|c_{0}\right|}^{2}\right)\sqrt{\kappa_{ce}}\varepsilon_{s}}{\Lambda_{1}\left(\Lambda_{2}^{*}-2ig^{2}\lambda_{1}{\left|c_{0}\right|}^{2}\right)-2ig^{2}\Lambda_{2}^{*}\lambda_{1}{\left|c_{0}\right|}^{2}},$$

where $\Lambda_{1}=-i\Delta^{{}^{\prime }}+\kappa_{c}-i\Omega +iJ\eta_{1}$, $\Lambda_{2}=i\Delta^{{}^{\prime }}+\kappa_{c}+i\Omega +iJ\eta_{2}$, $\eta_{1}=-iJ/\left(i\Delta_{a}+\kappa_{a}-i\Omega \right)$, $\eta_{2}=-iJ/\left(i\Delta_{a}+\kappa_{a}+i\Omega \right)$, $\lambda_{1}=\omega_{m}/\left(\omega_{m}^{2}-i\gamma_{m}\delta -\Omega^{2}\right)$.

According to the standard input–output relation [57] $c_{out}\left(t\right)=\varepsilon_{s}\left(t\right)-\sqrt{2\kappa }c\left(t\right)$ (where $c_{out}\left(t\right)$ is the output field operator), the transmission rate of the probe field is defined as [30,31,32,33,34,35,36]:

(10) $$T={\left|t(\omega_{s})\right|}^{2}={\left|\frac{c_{out}\left(t\right)}{\varepsilon_{s}\left(t\right)}\right|}^{2}=\left|1-\frac{\sqrt{\kappa_{ce}}c_{+}}{\varepsilon_{s}}\right|.$$

In order to investigate the group delay, we introduced group delay $\tau_{g}$ which is defined by:

(11) $$\tau_{g}=\frac{d\phi_{t}}{d\omega_{s}}{{|}_{\omega_{s=\omega_{p}}}=\frac{d\{\arg\left[t\left(\omega_{s}\right)\right]\}}{d\omega_{s}}|}_{\omega_{s=\omega_{p}}},$$

where $\phi_{t}=\arg\left[t\left(\omega_{s}\right)\right]$ is the phase dispersion playing a key role in the coherent optical propagation. The positive group delay, i.e., $\tau_{g}>0$, means the fast light, while the negative delay group, i.e., $\tau_{g}<0$, denotes the slow light, respectively.

## 3. Numerical Results and Discussion

The parameters used in this paper were [32]: $g_{0}/2\pi =12$ GHz/nm, $\gamma_{m}/2\pi =41$ kHz, $\omega_{m}/2\pi =51.8$ MHz, $\kappa_{c}/2\pi =\kappa_{a}/2\pi =15$ MHz, P=4 μW, the effective mass M=20 ng, the wave length of the laser $\lambda_{0}=750$ nm, and the coupling strength [53] $J∼\sqrt{\kappa_{c}\kappa_{a}}$. As we know, the optomechanical coupling between the mechanical mode and optical mode [32] induced by the phenomenon of OMIT was observed and the OMIT-induced slow light effect was also investigated [33]. When another optical cavity was introduced to the optomechanical system to form a photonic molecule optomechanics [56], we demonstrated that the tunable OMIT could realize the conversion from slow to fast light by controlling the coupling strength of the two optical cavities *J*. Here, in this paper, we considered the fixed optomechanical coupling rate *g* and unchanged coupling strength *J* of the two cavities, and we investigated the coherent phonon pump to the mechanical mode and the decay rate ratio parameter δ of the two cavities that influenced the OMIT and OMIT-induced slow light effect.

**Figure 2:**
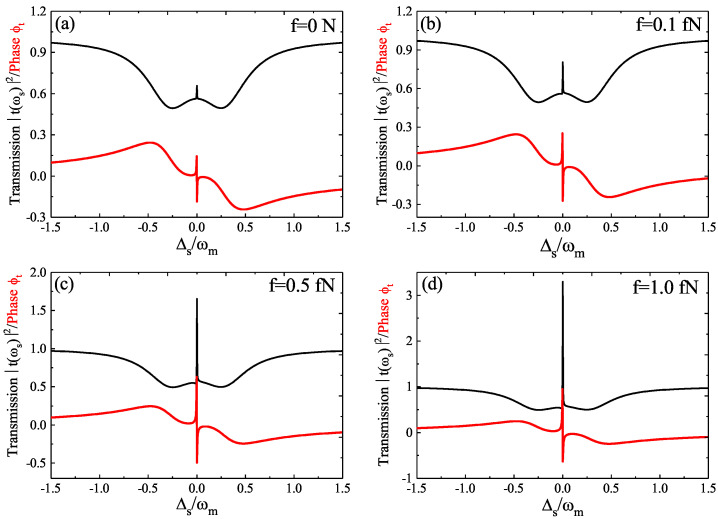
The transmission *T* (black curve) and phase $\phi_{t}$ (red curve) of the probe light as a function of $\Delta_{s}$ for four different driving amplitude *f*: (**a**) f=0; (**b**) f=0.1 fN; (**c**) f=0.5 fN; (**d**) f=1.0 fN. The other parameters are $J=1.0\kappa_{c}$, $\varphi_{m}=\pi /2$, P=5 μW, and $\Delta_{c}=\Delta_{a}=\omega_{m}$.

Figure 2 plots the transmission *T* (i.e., the black curve) and the phase $\phi_{t}$ (i.e., the red curve) of the probe field as a function of the probe–cavity detuning $\Delta_{s}=\omega_{s}-\omega_{c}$ under fixed *g* and $J=1.0\kappa_{c}$ for four different driving amplitudes *f* of the phonon driving with the pump phase $\varphi_{m}=\pi /2$ in the condition of $\Delta_{c}=\Delta_{a}=\omega_{m}$. In Figure 2a, the driving amplitude *f* of the phonon pump was f=0, and we can see that the line profile of transmission *T* shows a mode splitting due to the existence of the coupling strength *J*, and there is also a small transparency window at $\Delta_{s}=0$ due to the optomechanical coupling *g*. However, when the driving amplitude f≠0, with increasing the driving amplitude *f* from f=0.1 fN to f=1.0 fN as shown in Figure 2b–d, we found that the transparency window at $\Delta_{s}=0$ enhanced, i.e., if considering the phonon driving, the transmission of the OMIT window even exceeded unity and reached amplification. The above phenomena can be explained as follows: When there is no phonon pump in the system, the radiation pressure force coming from the pump laser field on the WGM cavity c applies to the mechanical mode changing the mechanical displacement of the phonon mode, which alters the frequency $\omega_{c}$ of the optomechanical cavity c; as a result, the mechanical mode resonates near its coherent oscillation frequency in the condition of $\Delta_{c}=\omega_{m}$. Once the beat frequency $\Omega =\omega_{s}-\omega_{p}$ (i.e., the probe-pump detuning) is close to the mechanical mode frequency $\omega_{m}$, the phonon mode starts to oscillate coherently, which leads to the Stokes frequency ($\Delta_{S}=\omega_{p}-\omega_{m}$) and anti-Stokes frequency ($\Delta_{AS}=\omega_{p}+\omega_{m}$) from the pump field. Due to the system being driven at $\Delta_{c}=\omega_{m}$, only the anti-Stokes frequency builds up the cavity and the Stokes frequency is suppressed; as a result, the destructive interference between the anti-Stokes frequency and the probe field modify the transmission spectrum, which manifests the OMIT window. When the weak coherent phonon pump is taken into consideration, the coupling between the mechanical mode and photon mode is enhanced due to the mechanical mode being driven by the phonon pump, which induces the enhanced interference effects; as a result, the OMIT window can even exceed unity as shown in Figure 2c,d. The phase $\phi_{t}$ of the probe field also changes significantly by increasing the driving amplitude *f*. Then, we studied the driving amplitude *f*-induced slow light effect.

**Figure 3:**
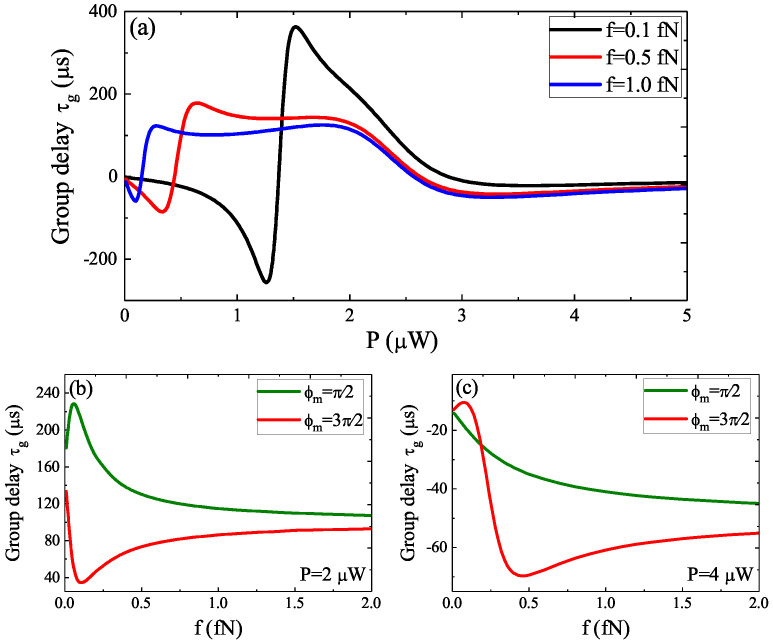
(**a**) The group delay $\tau_{g}$ as a function of optical pump power *P* for three driving amplitudes *f*. (**b**) The group delay $\tau_{g}$ versus *f* for two different pump phases $\varphi_{m}$ at P=2 μW. (**c**) The group delay $\tau_{g}$ versus *f* for two different pump phases $\varphi_{m}$ at P=4 μW.

In Figure 3a, we gave the group delay $\tau_{g}$ as a function of the optical pump power *P* for three driving amplitudes *f* under the parameters of $\varphi_{m}=\pi /2$ and $\Delta_{c}=\Delta_{a}=\omega_{m}$, and we could obtain that the evolution process of the group delay $\tau_{g}$ varied acutely, which experienced the conversion from $\tau_{g}<0$ to $\tau_{g}>0$. Especially, when the pump power P< 3 μW, the group delay $\tau_{g}$ presents the conversion from fast light to slow light, while the fast light dominates when P> 3 μW as shown in Figure 3a. In Figure 3b, we further plotted the group delay $\tau_{g}$ versus the driving amplitude *f* for two different pump phases $\varphi_{m}$ of the phonon driving at the optical pump power P=2 μW, and it was obvious that $\tau_{g}$ manifested the slow light effect. However, the difference was that the group delay $\tau_{g}$ firstly reached to a maximum value and then reduced to a constant value by increasing *f* for $\varphi_{m}=\pi /2$, and if $\varphi_{m}=3\pi /2$, the group delay $\tau_{g}$ firstly reached a minimum value and then increased to a constant value by increasing *f*. However, when we increased the optical pump power *P* to P=4 μW, the group delay $\tau_{g}$ was different from the case of P=4 μW. In Figure 3c, we found that the group delay $\tau_{g}$ decreased progressively and then reached a constant value at $\varphi_{m}=\pi /2$, while for $\varphi_{m}=3\pi /2$, the group delay $\tau_{g}$ firstly reached a maximum value and then reduced to a minimum value and finally reached a constant. Obviously, the driving amplitude *f* and the pump phase $\varphi_{m}$ of the phonon driving together influenced the fast and slow lights.

**Figure 4:**
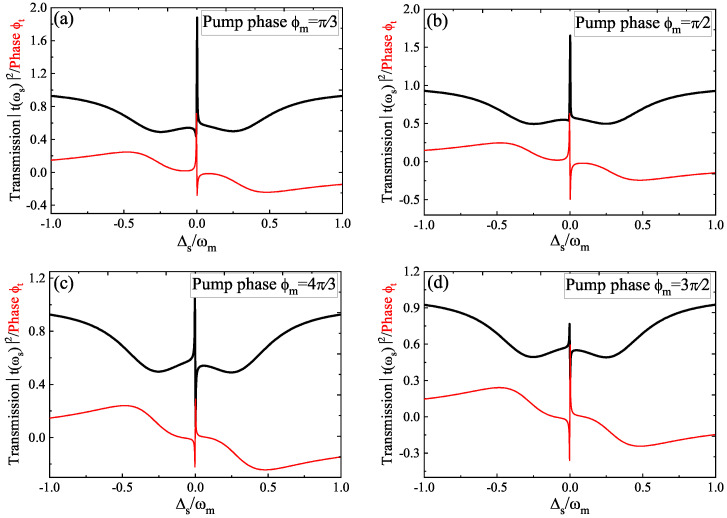
The transmission *T* and phase $\phi_{t}$ as a function of $\Delta_{s}$ for four different pump phases $\varphi_{m}$: (**a**) $\varphi_{m}=\pi /3$; (**b**) $\varphi_{m}=\pi /2$; (**c**) $\varphi_{m}=4\pi /3$; (**d**) $\varphi_{m}=3\pi /2$.

Then, in the following, we further studied the two parameters of the driving amplitude *f* and the pump phase $\varphi_{m}$ of the phonon driving that affected the OMIT and the slow light effect. In Figure 4, we displayed the transmission *T* (i.e., the black curve) and the phase $\phi_{t}$ (i.e., the red curve) versus $\Delta_{s}$ for four different pump phases $\varphi_{m}$ at the driving amplitude f=0.5 fN of the phonon driving. We found that the transparency window around $\Delta_{s}=0$ exceeded unity at $\varphi_{m}=\pi /3$; with increasing the pump phase $\varphi_{m}$ to $\varphi_{m}=4\pi /3$, the intensity of the transparency window was reduced, and when $\varphi_{m}$ reached $\varphi_{m}=3\pi /2$, the transparency window fell below the unity. Therefore, the transparency window underwent the conversion from amplification to transparency by increasing the pump phase $\varphi_{m}$ of the phonon driving. The phase $\phi_{t}$ of the probe field also changes significantly for increasing the pump phase $\varphi_{m}$ of the phonon driving. Then, we studied the pump phase $\varphi_{m}$-induced slow light effect.

**Figure 5:**
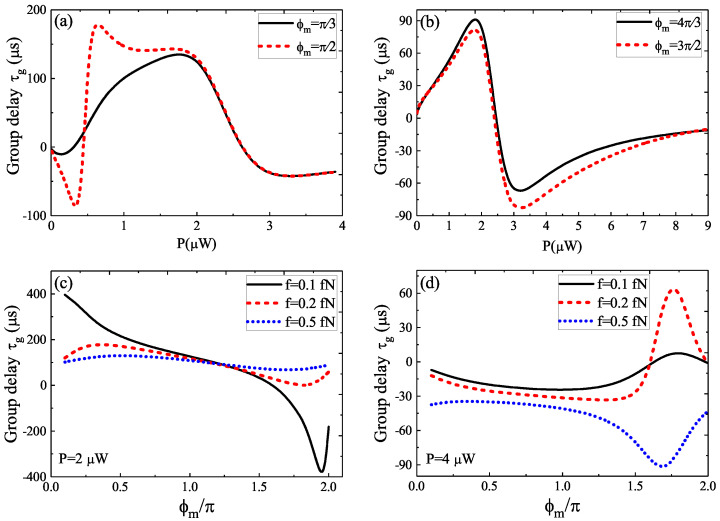
(**a**) The $\tau_{g}$ as a function of *P* for $\varphi_{m}=\pi /3$ and $\varphi_{m}=\pi /2$. (**b**) The $\tau_{g}$ as a function of *P* for $\varphi_{m}=4\pi /3$ and $\varphi_{m}=3\pi /2$. (**c**) The $\tau_{g}$ as a function of $\varphi_{m}$ for three driving amplitudes *f* at P=2 μW. (**d**) The $\tau_{g}$ as a function of $\varphi_{m}$ for three driving amplitudes *f* at P=4 μW.

Figure 5a plots the group delay $\tau_{g}$ versus the optical pump power *P* for two different pump phases of $\varphi_{m}=\pi /3$ and $\varphi_{m}=\pi /2$ at f=0.5 fN. The results indicated that the group delay $\tau_{g}$ experienced the conversion of $\tau_{g}<0$ to $\tau_{g}>0$ at both $\varphi_{m}=\pi /3$ and $\varphi_{m}=\pi /2$, i.e., the conversion from fast to slow light. However, the processes of evolution were different at P<2 μW, and at P>2 μW the group delay $\tau_{g}$ converged. In Figure 5b, we considered another two phases of $\varphi_{m}=4\pi /3$ and $\varphi_{m}=3\pi /2$, and we found that the group delay $\tau_{g}$ experienced the same process of conversion from fast to slow light, i.e., the group delay $\tau_{g}$ firstly reached a maximum value and then reduced to a minimum value and finally reached a constant. On the other hand, we also showed the group delay $\tau_{g}$ as a function of the pump phase $\varphi_{m}$ for three different driving amplitudes *f* at a fixed pump power P=2 μW as shown in Figure 5c. When f=0.1 fN, $\tau_{g}$ experienced $\tau_{g}>0$ to $\tau_{g}<0$ by increasing the pump phase $\varphi_{m}$ from $\varphi_{m}=0$ to $\varphi_{m}=2\pi$. When f=0.2 fN or f=0.5 fN, the group delay $\tau_{g}>0$ was dominating and, interestingly, the group delay $\tau_{g}$ showed mirror antisymmetry and the axis of symmetry was $\varphi_{m}=\pi$. In Figure 5d, we further plotted the group delay $\tau_{g}$ versus $\varphi_{m}$ for three different *f* at P=4 μW, which was very different from the case of P=2 μW. We saw that the group delay $\tau_{g}$ experienced the conversion from fast to slow light by increasing $\varphi_{m}$ from $\varphi_{m}=0$ to $\varphi_{m}=2\pi$ at f=0.1 fN and f=0.2 fN. When the driving amplitude *f* reached f=0.5 fN, the group delay $\tau_{g}$ only manifested the fast light.

**Figure 6:**
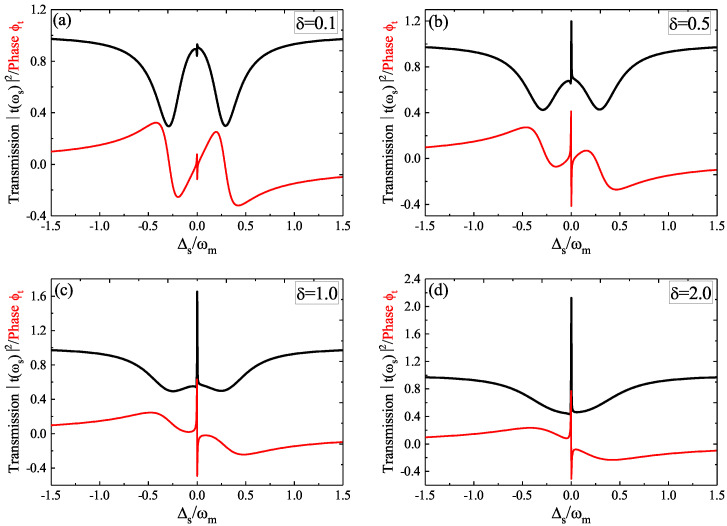
The transmission *T* and phase $\phi_{t}$ as a function of $\Delta_{s}$ for four ratio parameters δ: (**a**) δ=0.1; (**b**) δ=0.5; (**c**) δ=1.0; (**d**) δ=2.0.

**Figure 7:**
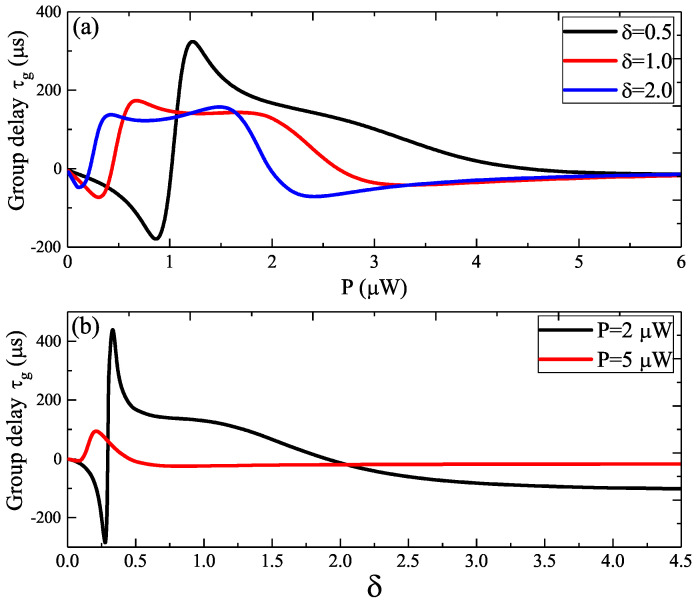
(**a**) The $\tau_{g}$ as a function of *P* for three δ. (**b**) The group delay $\tau_{g}$ as a function of δ for two *P*.

On the other hand, it was hard to reach the high quality factor (Q) and small volume (V) concurrently in the same cavity mode due to the diffraction limit. The smaller V means a larger radiative decay rate leading to a lower Q. Although different types of cavities control their own unique properties, the competition of a high Q and small V still exists. If the optomechanical cavity c with a high cavity dissipation was coupled to an auxiliary cavity a with a high Q but a large V, the diffraction limit could be changed. Here, we used a ratio parameter $\delta =\kappa_{a}/\kappa_{c}$ ($\kappa_{c}=\omega_{c}/Q_{c}$ and $\kappa_{a}=\omega_{a}/Q_{a}$, where $Q_{c}$ and $Q_{a}$ are the Q of the two optical cavities) and studied the parameter that affected the OMIT. In Figure 6, we showed the transmission *T* and the phase $\phi_{t}$ versus $\Delta_{s}$ for four different δ at the parameters of the driving amplitude f=0.5 fN, the pump phase $\varphi_{m}=\pi /2$, the optical pump power P=5 μW, and $J=1.0\kappa_{c}$. In Figure 6a, we considered δ=0.2, i.e., $\kappa_{a}=0.2\kappa_{c}$ which means $Q_{a}>Q_{c}$, transmission *T* showing a remarkable mode splitting due to the existence of the cavity–cavity coupling *J*, and the optomechanical coupling-induced transparency window was not obvious. With increasing the ratio parameter δ from δ=0.5 to δ=2.0, we found the OMIT window was enhanced (even exceeded the unity) due to the role of the phonon driving-induced strong optomechanical coupling while the mode splitting behaviour (appearing in Figure 6a) induced by the parameter *J* was reduced. Thus, by flexibly designing the parameters of the two cavities in the photonic-molecule optomechanics, such as the photon decay rate κ or the quality factors of the two cavities, the transmission *T* can be controlled easily in the photonic-molecule optomechanical system. The phase $\phi_{t}$ of the probe field also changed significantly for the ratio parameter δ and, then, we studied the parameter δ that influenced the slow light effect in the following:

In Figure 7a, we plotted the group delay $\tau_{g}$ versus the optical pump power *P* for three different ratio parameters δ under the parameters of f=0.5 fN and $\varphi_{m}=\pi /2$. The results indicated that the group delay $\tau_{g}$ experienced the conversion from fast to slow light, while the difference was that the process of evolution of the group delay $\tau_{g}$ in the condition of δ≥1 was more complicated than in the condition of δ<1. In Figure 7b, we also gave the group delay $\tau_{g}$ as a function of δ for two optical pump powers *P*. We found that in the condition of P=2 μW, the group delay $\tau_{g}$ experienced the process of $\tau_{g}<0$ to $\tau_{g}>0$, while if P=5 μW, the slow light effect was dominating. Therefore, we could control the Q of the two cavities to reach the conversion from fast to slow light.

## 4. Conclusions

In conclusion, we theoretically demonstrated the optical response properties in a photonic-molecule optomechanical system with driving by a strong pump field, a weak probe field, and phonon driving. The numerical simulations showed that an enhancement OMIT, i.e., the probe transmission, could exceed unity and could be obtained by controlling the driving amplitude and pump phase of the phonon pump. In addition, the phase dispersion of the transmitted probe field could also be modulated, which leads to the tunable conversion from the slow light to fast light effect. Finally, the numerical simulations indicated that the group delay of the transmitted probe field could be controlled by tuning several parameters, which includes the power of the pump field, the ratio parameter of the two cavities, and the driving amplitude and pump phase of the phonon driving, even reaching a conversion between the slow and fast light effect in the photonic-molecule optomechanics.

## Data Availability

Not applicable.
